# Induction of heme oxygenase-1 attenuates lipopolysaccharide-induced cyclooxygenase-2 expression in mouse brain endothelial cells

**DOI:** 10.1186/1742-2094-7-86

**Published:** 2010-11-30

**Authors:** Ruey-Horng Shih, Chuen-Mao Yang

**Affiliations:** 1 Department of Physiology and Pharmacology, Chang Gung University, Kwei-San, Tao-Yuan, Taiwan

## Abstract

**Background:**

Prostaglandin E_2 _(PGE_2_), an arachidonic acid metabolite converted by cyclooxygenase-2 (COX-2), plays important roles in the regulation of endothelial functions in response to bacterial infection. The enzymatic activity of COX-2 can be down-regulated by heme oxygenase-1 (HO-1) induction. However, the mechanisms underlying HO-1 modulating COX-2 protein expression are not known.

**Objective:**

The aim of the present study was to investigate whether the up-regulation of HO-1 regulates COX-2 expression induced by lipopolysaccharide (LPS), an endotoxin produced by Gram negative bacteria, in mouse brain endothelial cells (bEnd.3)

**Methods:**

Cultured bEnd.3 cells were used to investigate LPS-induced COX-2 expression and PGE_2 _production. Cobalt protoporphyrin IX (CoPP, an HO-1 inducer), infection with a recombinant adenovirus carried with HO-1 gene (Adv-HO-1), or zinc protoporphyrin (ZnPP, an HO-1 inhibitor) was used to stimulate HO-1 induction or inhibit HO-1 activity. The expressions of COX-2 and HO-1 were evaluated by western blotting. PGE_2 _levels were detected by an enzyme-linked immunoassay. Hemoglobin (a chelator of carbon monoxide, CO, one of metabolites of HO-1) and CO-RM2 (a CO releasing molecule) were used to investigate the mechanisms of HO-1 regulating COX-2 expression.

**Results:**

We found that LPS-induced COX-2 expression and PGE_2 _production were mediated through NF-κB (p65) via activation of Toll-like receptor 4 (TLR4). LPS-induced COX-2 expression was inhibited by HO-1 induction by pretreatment with CoPP or infection with Adv-HO-1. This inhibitory effect of HO-1 was reversed by pretreatment with either ZnPP or hemoglobin. Pretreatment with CO-RM2 also inhibited TLR4/MyD88 complex formation, NF-κB (p65) activation, COX-2 expression, and PGE_2 _production induced by LPS.

**Conclusions:**

We show here a novel inhibition of HO-1 on LPS-induced COX-2/PGE_2 _production in bEnd.3. Our results reinforce the emerging role of cerebral endothelium-derived HO-1 as a protector against cerebral vascular inflammation triggered by bacterial infection.

## Background

Sepsis is a life-threatening clinical syndrome which is correlated with a mortality of 30% [1]. During Gram negative sepsis, lipopolysaccharide (LPS), an endotoxin produced by Gram negative bacteria, stimulates various pro-inflammatory cytokines and mediators releasing [2]. For example, prostaglandin E_2 _(PGE_2_), an arachidonic acid metabolite synthesized by cyclooxygenase-2 (COX-2) which is a key enzyme involved in the LPS-induced inflammatory process [3], is a potent pro-inflammatory mediator and plays important physiological/pathological roles in the regulation of vascular endothelial function [4,5].

LPS may reach the brain tissue via blood stream and induce neuronal injury during bacterial infection [6]. LPS-induced brain inflammation was closely associated with increased oxidative stress [7,8]. Therefore, LPS has been extensively used to study the possible linkage between inflammation and brain injury [9]. In the brain, cerebrovascular endothelial damage induced by oxidative stress have been reported to be diminished by heme oxygenase-1 (HO-1) [10-12], an inducible form of HO which is an enzyme catalyzing the degradation of heme into carbon monoxide (CO), biliverdin, and free iron. HO-1 is one of the major acute-phase proteins up-regulated upon exposure to oxidative stresses [13-15]. Moreover, increasing evidence indicates that CO, a by-product of HO-1, may protect against LPS-induced endothelial injury, suggesting that the production of CO by HO-1 exerts protective effects against LPS-induced endothelial injury [16].

COX-2 is an enzymatic heme protein [17]. HO-1 controls the availability of heme for synthesis of enzymatic heme proteins (e.g. COX-2), and generates CO, which binds the heme moiety of heme protein thus affecting their enzymatic activity [18]. In consequence, the effect of HO-1 on COX-2 protein in vascular endothelial cells is one of significant physiological/pathological modulation. However, there are few studies investigating whether the up-regulation of COX-2 protein can be modulated by HO-1. The effect of HO-1 on LPS-induced COX-2 expression in cerebral endothelial cells has not yet been elucidated.

Thus, experiments were performed using a mouse brain endothelial cell line (bEnd.3) to investigate whether HO-1 regulates LPS-induced COX-2 expression in these cells. Our results demonstrate that LPS-induced COX-2 expression and PGE_2 _production is inhibited by induction of HO-1. This inhibitory effect of HO-1 is mediated through an HO-1 byproduct, CO, which attenuates TLR4/MyD88 complex formation and NF-κB activation induced by LPS. These results suggest that cerebrovascular endothelium-derived HO-1 may prevent vascular inflammation which is triggered by bacterial infection in the brain.

## Methods

### Materials

Dulbecco's modified Eagle's medium (DMEM) and fetal bovine serum (FBS) were purchased from Invitrogen (Carlsbad, CA, USA). Polyclonal antibodies HO-1, TLR4, and MyD88 were purchased from Santa Cruz (Santa Cruz, CA, USA). Anti-COX-2 antibody was purchased from BD Transduction Laboratories (San Diego, CA, USA). Anti-GAPDH antibody was purchased from Biogenesis (Boumemouth, UK). Anti-phospho-NF-κB p65 antibody was purchased from Cell Signaling (Danver, MA, USA). Bay117082 was purchased from Biomol (Plymouth Meeting, PA, USA). Bicinchoninic acid (BCA) protein assay reagent was purchased from Pierce (Rockford, IL, USA). Hemoglobin, biliverdin reductase, enzymes, and other chemicals were purchased from Sigma (St. Louis, MO, USA).

### Cell cultures

Mouse brain endothelial cells (bEnd.3, ATCC CRL-2299) were grown in DMEM/F-12 containing 10% FBS and antibiotics (100 U/ml penicillin G, 100 μg/ml streptomycin, and 250 ng/ml fungizone) at 37°C in a humidified 5% CO_2 _atmosphere. When the cultures grew to confluence (about 4 days), cells were detached with 0.05% (w/v) trypsin/0.53 mM EDTA for 5 min at 37°C. The cell suspension was diluted with DMEM/F-12 containing 10% FBS to a concentration of 2 × 10^5 ^cells/ml. The cell suspension was plated onto 12-well culture plates (1 ml/well) for the measurement of protein expression and enzymatic assays. Culture medium was changed after 24 h and every 3 days. Experiments were performed with cells from passages 5 to 13.

### Preparation of recombinant adenovirus infection

A recombinant adenovirus containing HO-1 (Adv-HO-1) was kindly provided by Dr. L.Y. Chau (Institute of Biomedical Sciences, Academia Sinica, Taipei, Taiwan). Recombinant adenovirus was generated by homologous recombination and amplified in 293 cells. Large scales of viral vectors were purified by CsCl ultracentrifugation and stored in 10 mM Tris-HCl (pH 7.4), 1 mM MgCl_2_, and 10% (v/v) glycerol at -80°C until used for experiments. Virus titers were determined by a plaque assay on a 293 cell monolayer. The recombinant adenovirus was diluted with DMEM/F12 medium and added directly to the cells (MOI = 10). After 24 h of infection, the cells were incubated with LPS for another 24 h. Cell lysates were analyzed by western blotting.

### Cell extract preparation and western blot

Western blot analysis was performed as previously described [19]. bEnd.3 cells were lysed with a sample buffer (125 mM Tris-BASE, 5% Glycerol, 3% β-mercaptoethanol, 1.25% SDS and 0.0005% Bromophenol blue). Cell lysates were denatured, subjected to SDS-PAGE using a 12% running gel and transferred onto a nitrocellulose membrane. Membranes were incubated with a mouse anti-COX-2 antibody (1:1000 dilution) or a goat anti-HO-1 antibody (1:1000 dilution) at 4°C for 24 h, and then incubated with an anti-mouse or anti-goat horseradish peroxidase antibody (1:2000 dilution, at room temperature for 1 h), respectively. The gel bands were detected by ECL reagents and were quantified by a densitometry.

### Immunoprecipitation assay

The bEnd.3 cells were grown to confluence and starved for 24 h in serum-free DMEM/F-12 medium. Cells were pretreated with CO-RM2 (CO releasing molecule, 50 μM) for 2 h prior to the application of LPS. The cells were washed, scraped, and centrifuged to prepare membrane, cytosolic, and nuclear fractions, as previously described [20]. Membrane fractions containing 1 mg of protein were incubated with 2 μg of anti-TLR4 antibody at 4°C for 24 h, and then 10 μl of 50% protein A-agarose beads was added and mixed at 4°C for another 24 h. The immunoprecipitates were collected and subjected to electrophoresis on 12% SDS-PAGE, transferred to nitrocellulose membrane, and then blotted using an anti-MyD88 or anti-TLR4 antibody. The gel bands were detected by ECL reagents and were quantified by a densitometry.

### Enzymatic assay for HO-1 activity

HO activity was measured as the level of bilirubin formation using the microsomal fraction of cells [21]. Briefly, bEnd.3 cells were washed twice with PBS, gently scraped off the dish, and centrifuged (1000 ×g, 10 min, 4°C). The cell pellet was suspended in MgCl_2 _(2 mM) and phosphate (100 mM) buffer (pH 7.4), frozen at -70°C, thawed thrice, and finally sonicated on ice before centrifugation at 18,000 ×g for 10 min at 4°C. The supernatant (400 μl) was added to a reaction mixture (200 μl final volume, pH 7.4) containing NADPH (0.8 mM), glucose-6-phosphate (2 mM), glucose-6-phosphate-1-dehydrogenase (0.2 U), and 2 mg of rat liver cytosol as a source of biliverdin reductase, PBS (100 mM), and the substrate hemin (10 μM). The reaction was conducted for 1 h at 37°C in the dark and terminated by addition of 1 ml chloroform. The reaction without the NADH served as a control. The extracted bilirubin was measured by the difference in absorption between 464 and 530 nm (extinction coefficient = 40 mM^-1^·cm^-1^) with a spectrophotometer. HO activity was expressed as picomoles of bilirubin per milligram of protein per hour.

### Measurement of PGE_2 _generation

bEnd.3 cells were cultured in 12-well culture plates. After reaching confluence, cells were pretreated with CoPP, ZnPP, or CO-RM2 for the indicated time intervals, and then incubated with LPS (100 μg/ml) at 37°C for 24 h. The levels of PGE_2 _released into the culture medium were collected and stored at -80°C until being assayed by using a commercially available PGE_2 _enzyme immunoassay kit (Cayman Chemicals, Ann Arbor, MI). Both the samples and standards were assayed in parallel.

### Statistical analysis of data

Data were analyzed using a GraphPad Prism Program (GraphPad, San Diego, CA, USA). Quantitative data were analyzed using one-way analysis of variance (ANOVA) followed by Tukey's honestly significant difference tests between individual groups. Data were expressed as mean ± SEM. A value of *P *< 0.05 was considered significant.

## Results

### LPS induces COX-2 expression and PGE_2 _synthesis in bEnd.3 cells

**Figure 1:**
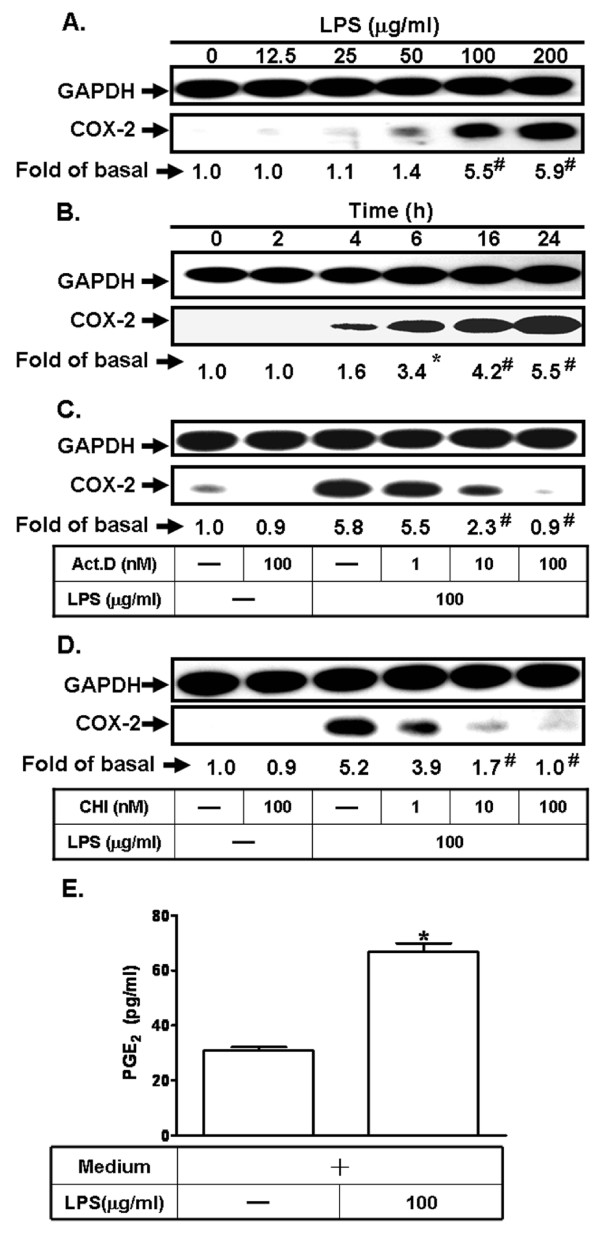
**LPS induces COX-2 protein expression and activity**. **A, B: **bEnd.3 cells were incubated with various concentrations of LPS for the indicated time intervals. The expression of COX-2 was determined by western blot.*P < 0.05; ^#^*P *< 0.01, as compared with the cells exposed to the vehicle. **C, D**: Cells were pretreated with either actinomycin D (Act.D) or cycloheximide (CHI) for 1 h, and then incubated with LPS for 24 h. The expression of COX-2 was determined by western blot. *P < 0.05; ^#^*P *< 0.01, as compared with the cells incubated with LPS alone. **E: **Cells were incubated with 100 μg/ml LPS for 24 h. PGE_2 _production was determined as the activity of COX-2. Data are summarized and expressed as mean ± SEM of four individual experiments. *P < 0.05, as compared with the cells exposed to the vehicle.

To determine the effect of LPS on COX-2 expression, bEnd.3 cells were incubated with various concentrations of LPS for the indicated time intervals. As shown in Figure 1A and 1B, LPS induced COX-2 expression in a time- and concentration-dependent manner. LPS (100 μg/ml)-induced COX-2 protein expression was significantly increased within 6 h and sustained over 24 h. To further determine if LPS-induced COX-2 expression required ongoing transcription or translation, cells were pretreated with either a transcriptional level inhibitor [actinomycin D (Act.D)] or a translational level inhibitor [cycloheximide (CHI)] for 1 h and then incubated with LPS (100 μg/ml) for 24 h. As shown in Figure 1C and 1D, LPS-mediated induction of COX-2 expression was abolished by pretreatment with either Act.D or CHI in a concentration-dependent manner. Pretreatment with these two inhibitors alone had no effect on COX-2 expression. In addition, we found that LPS (100 μg/ml) markedly increased COX-2 enzyme activity, revealed as PGE_2 _production (Figure 1E). Taken together, these findings demonstrated that the induction of COX-2 by LPS depends on *de novo *protein synthesis.

### LPS-induced COX-2 expression is mediated through NF-κB activation

**Figure 2:**
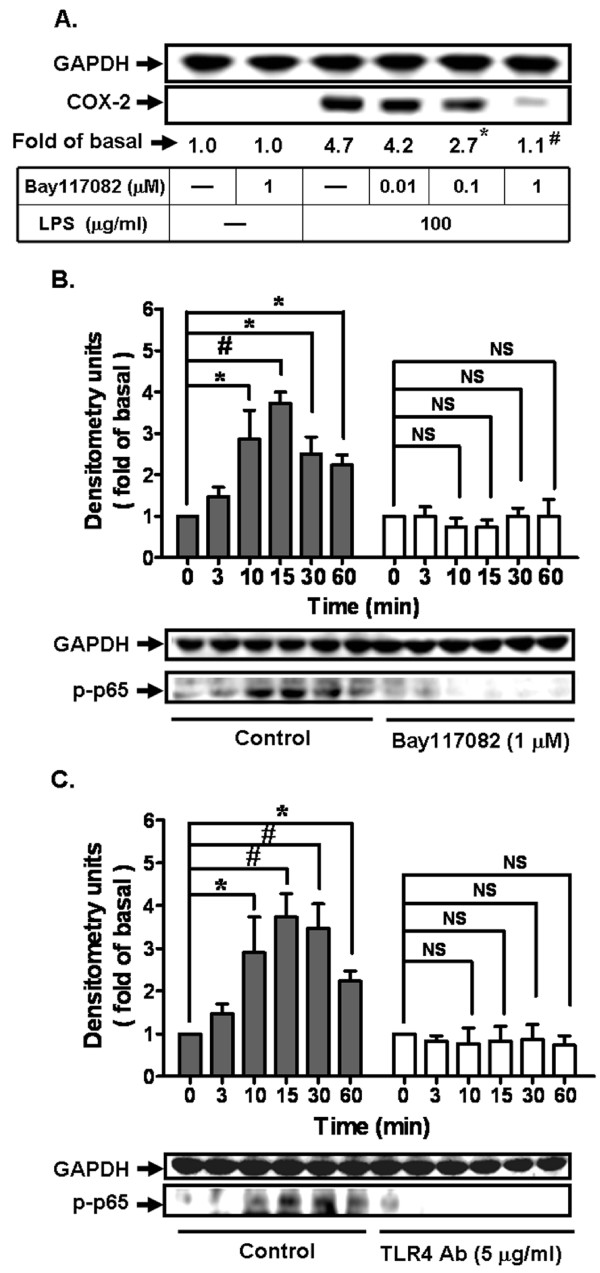
**LPS-induced COX-2 expression via NF-κB activation**. **A: **Cells were pretreated with an inhibitor of NF-κB, Bay117082, for 1 h and then incubated with LPS (100 mg/ml) for 24 h. The expression of COX-2 was determined by western blot. *P < 0.05; ^#^*P *< 0.01, as compared with the cells incubated with LPS alone. **B, C**: Cells were pretreated with either Bay117082 or TLR 4 antibody for 1 h and then stimulated with LPS (100 mg/ml) for the indicated time intervals. Phosphorylation of NF-κB p65 was determined by western blot. Data are summarized and expressed as mean ± SEM of four individual experiments. *P < 0.05; ^#^*P *< 0.01 as compared within groups. NS: not significant.

To investigate the role of NF-κB in LPS-mediated COX-2 expression, cells were pretreated with an inhibitor of NF-κB, Bay117082 for 1 h, and then incubated with LPS for 24 h. As shown in Figure 2A, Bay117082 markedly inhibited LPS-induced COX-2 expression, suggesting that NF-κB may play an important role in LPS-induced COX-2 expression. Next, we investigated whether the involvement of NF-κB in LPS-mediated responses was mediated through activation of NF-κB (p65). As shown in Figure 2B, LPS stimulated phosphorylation of NF-κB (p65) in a time-dependent manner, which was inhibited by pretreatment with Bay117082. To further investigate whether activation of NF-κB was mediated through TLR4 receptors, cells were pretreated with a TLR4 antibody (5 mg/ml) for 1 h, and then incubated with LPS for the indicated time intervals. As shown in Figure 2C, pretreatment with TLR4 antibody also inhibited LPS-stimulated phosphorylation of NF-κB (p65). These data indicated that LPS-induced COX-2 expression was mediated through a TLR4/NF-κB cascade in bEnd.3 cells.

### Pretreatment with CoPP attenuates LPS-induced COX-2 expression and NF-κB (p65) phosphorylation

**Figure 3:**
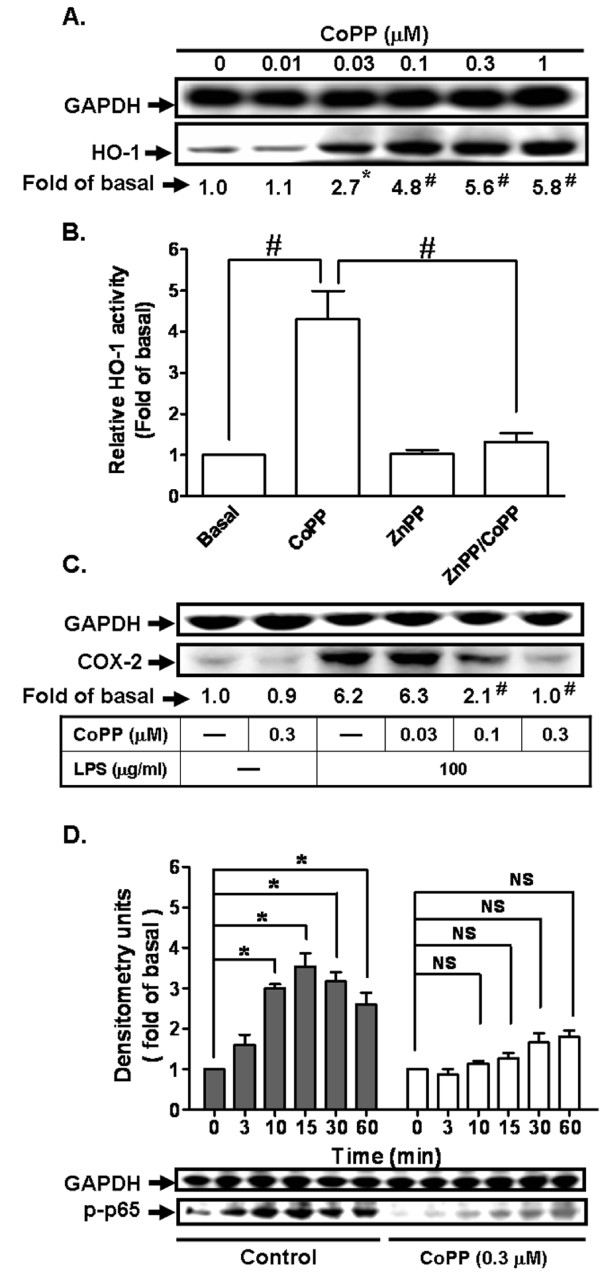
**LPS-induced COX-2 expression is attenuated by pretreatment with CoPP in bEnd.3 cells**. **A: **Cells were incubated with various concentrations of CoPP for 24 h. Expression of HO-1 was determined by western blot. *P < 0.05; ^#^*P *< 0.01 as compared the cells incubated with vehicle alone. **B: **Cells were pretreated with vehicle or ZnPP (0.1 μM) for 1 h and then incubated with CoPP (0.3 μM) for 24 h. HO-1 enzyme activity was determined. ^#^*P *< 0.01 as compared within groups. **C: **Cells were pretreated with various concentrations of CoPP for 24 h and then incubated with LPS (100 mg/ml) for 24 h. The expression of COX-2 was determined by western blot. *P < 0.05; ^#^*P *< 0.01, as compared with the cells incubated with LPS alone. **D: **Cells were pretreated with CoPP (0.3 μM) for 24 h and then stimulated with LPS (100 mg/ml) for the indicated time intervals. The phosphorylation of NF-κB p65 was determined by western blot. Data are summarized and expressed as mean ± SEM of four individual experiments. *P < 0.05; ^#^*P *< 0.01, as compared within groups. NS: not significant.

HO-1 has been shown to exert cytoprotective and anti-inflammatory effects in stress conditions. Therefore, we investigated whether induction of HO-1 attenuated LPS-mediated responses in bEnd.3 cells. As shown in Figure 3A, pretreatment with CoPP (an HO-1 inducer) induced HO-1 protein expression in a concentration-dependent manner in bEnd.3 cells. Pretreatment with CoPP (0.3 μM) for 24 h markedly increased HO-1 enzyme activity which was attenuated by ZnPP (an HO-1 activity inhibitor) (Figure 3B). Interestingly, pretreatment with CoPP resulted in a significant attenuation of LPS-induced COX-2 protein expression (Figure 3C). NF-κB is a crucial transcription factor for COX-2 expression, we determined the effect of HO-1 on LPS-stimulated NF-κB activation by measuring NF-κB (p65) phopshorylation. As shown in Figure 3D, LPS-stimulated NF-κB (p65) phosphorylation was also inhibited by pretreatment with CoPP (0.3 μM, 24 h), during the period of observation. These data indicated that LPS-induced COX-2 expression and NF-κB (p65) phosphorylation was attenuated by up-regulation of HO-1 in bEnd.3 cells.

### HO-1 over-expression attenuates LPS-induced COX-2 expression and PGE_2 _synthesis

**Figure 4:**
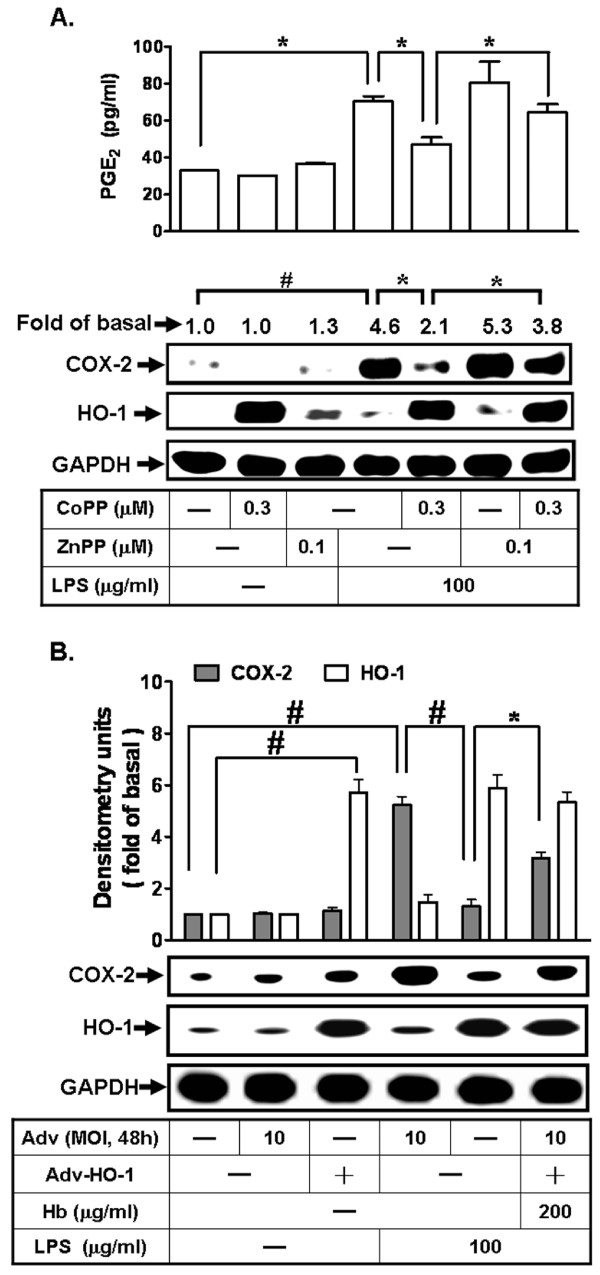
**Over-expression of HO-1 attenuates LPS-induced COX-2 expression and PGE_2 _synthesis in bEnd.3 cells**. **A: **Cells were pretreated with or without ZnPP for 1 h, incubated with CoPP for 24 h, and then incubated with LPS (100 mg/ml) for another 24 h. The media were used to determine the levels of PGE_2_. The expression of COX-2 and HO-1 were determined by western blot. **B: **Cells were pretreated with or without hemoglobin (Hb) for 1 h, infected with adenovirus (MOI = 10) or recombinant adenovirus carrying the human HO-1 gene treatment (Adv-HO-1, MOI = 10) for 48 h, and then incubated with LPS (100 mg/ml) for another 24 h. The levels of COX-2 and HO-1 expression were determined by western blot. Data are summarized and expressed as mean ± SEM for four individual experiments. *P < 0.05; ^#^*P *< 0.01 as compared within groups.

To ensure that HO-1 induction decreased LPS-induced COX-2 expression, bEnd.3 cells were pretreated with an HO-1 inducer (CoPP) or an HO-1 functional inhibitor (ZnPP). As shown in Figure 4A, pretreatment with CoPP attenuated LPS-induced COX-2 protein expression and PGE_2 _synthesis. These inhibitory effects of CoPP were reversed by ZnPP. To further confirm the effect of HO-1 overexpression induced by CoPP on LPS-mediated responses, bEnd.3 cells were transfected with either adenovirus or recombinant adenovirus carrying the human HO-1 (Adv-HO-1). As shown in Figure 4B, transfection with adv-HO-1 (Adv, MOI = 10, 48 h) enhanced HO-1 expression and attenuated LPS-induced COX-2 expression. In contrast, transfection with adenovirus alone had no effect on HO-1 and COX-2 experssion. This inhibitory effect was reversed by a chelator of carbon monoxide, hemoglobin (Hb, 200 mg/ml). These results suggested that overexpression of HO-1 by pretreatment with CoPP or transfection with adv-HO-1 attenuated LPS-induced COX-2 expression, at least in part, mediated through an HO-1 byproduct, CO.

### CO inhibits LPS-induced COX-2 expression and PGE_2 _synthesis in bEnd.3 cells

**Figure 5:**
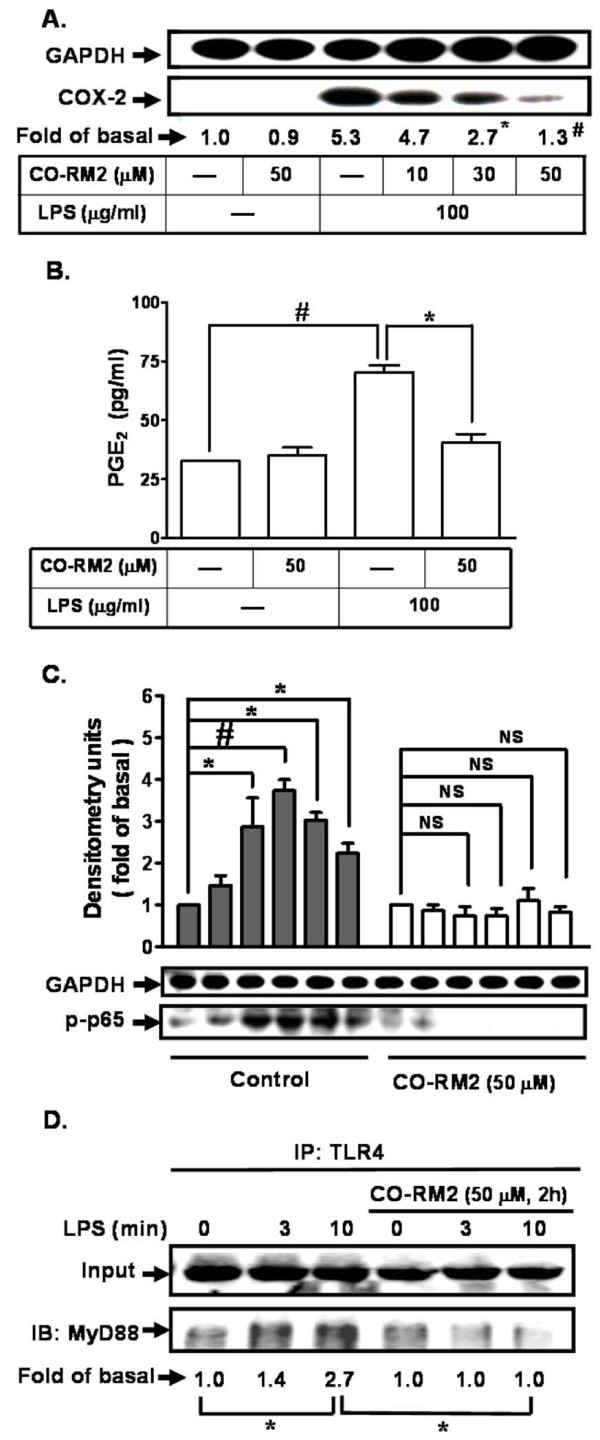
**CO inhibits LPS-induced COX-2 protein expression and PGE_2 _synthesis in bEnd.3 cells**. **A: **Cells were pretreated with CO-RM2 (CO releasing molecule) for 2 h and then incubated with LPS (100 mg/ml) for 24 h. The expression of COX-2 was determined by western blot. *P < 0.05; ^#^*P *< 0.01, as compared with the cells incubated with LPS alone. **B: **Cells were pretreated with CO-RM2 (50 mM, 2 h) and then incubated with LPS (100 mg/ml) for 24 h. The media were used to determine the levels of PGE_2_. *P < 0.05; ^#^*P *< 0.01, as compared within groups. **C: **Cells were pretreated with CO-RM2 (CO releasing molecule) for 2 h and then incubated with LPS (100 mg/ml) for the indicated time intervals. Phosphorylation of NF-κB p65 was determined by western blot. *P < 0.05; ^#^*P *< 0.01, as compared within groups. NS: not significant. **D: **Cells were treated with or without CO-RM2 (50 μM, 2 h) followed by LPS treatment (100 mg/ml) for the indicated time intervals. Cell lysates were immunoprecipitated with an anti-TLR4 antibody. The immunoprecipiates were analyzed by western blot using anti-MyD88 or anti-TLR4 (input) antibody. Data are summarized and expressed as mean ± SEM for four individual experiments. *P < 0.05 as compared within groups.

To further determine whether CO attenuated LPS-induced COX-2 expression, bEnd.3 cells were pretreated with a CO releasing molecule (CO-RM2). As shown in Figure 5A, LPS-induced COX-2 protein expression was inhibited by pretreatment with CO-RM2 in a concentration manner. Pretreatment with CO-RM2 also attenuated LPS-induced PGE_2 _synthesis (Figure 5B). We further determined whether the inhibitory effect of CO on LPS-induced responses was due to the attenuation of NF-κB activation. As shown in Figure 5C, LPS-stimulated NF-κB (p65) phosphorylation was also inhibited by pretreatment with CO-RM2.

LPS has been shown to activate NF-κB mediated through TLR4 leading to the expression of COX-2 [22]. We therefore investigated whether CO regulated LPS-induced COX-2 expression through interruption of either NF-κB or TLR4 in these cells. We tested the effect of CO-RM2 on LPS-induced protein-protein interaction between TLR4 and its adaptor protein myeloid differentiation factor (MyD88) which was shown to initiate an early activation of NF-κB in endothelial cells [23]. As shown in Figure 5D, pretreatment with CO-RM2 inhibited LPS-induced TLR4/MyD88 complex formation in bEnd.3 cells, indicating that CO attenuated the protein-protein interaction between TLR4 and MyD88 and thus retardation of COX-2 expression induced by LPS. These data suggested that LPS-stimulated TLR4/MyD88/NF-κB activation and COX-2 expression was blocked by CO in bEnd.3 cells.

## Discussion

PGs secreted by inflammatory cells are important in early initiation of inflammatory responses. PGE_2 _is thought to be a mediator of inflammation and pain [4,5]. COX-2, a key enzyme catalyzing the rate-limiting step in the inducible production of PGs, has been suggested to contribute to cellular damage. Therefore, COX-2 inhibition is suggested to be beneficial for cellular survive. In the present study, LPS-induced COX-2 expression and PGE_2 _synthesis was inhibited by HO-1 induction by pretreatment with either CoPP or Adv-HO-1 in murine bEnd.3 cells. Some studies also demonstrated an inhibitory effect of HO-1 on PGs production. Up-regulation of the HO-1 gene or pretreatment with an HO-1 inducer, SnCl_2_, attenuated angiotensin II-induced COX-2 and PG synthesis in endothelial cells [24]. Pretreatment with CoPP decreased PGE_2 _production by inhibiting microsomal prostaglandin E synthase-1 expression in primary cultured chondrocytes [25]. These data suggest that overexpression of HO-1 exerts an inhibitory effect on COX-2 expression and PGs production in different cell types. In contrast, an HO-1 inducer, CKD712, showed a weak inhibitory effect on COX-2 and PGE_2 _induced by LPS in RAW264.7 cells [26]. Therefore, the effect of HO-1 on expression of inflammatory proteins and mediators may be controversial. In the present study, our results are the first report to show that induction of HO-1 attenuates LPS-induced COX-2 expression and PGE_2 _synthesis in endothelial cells. Cerebral vascular endothelium-derived HO-1 may contribute to the prevention of vascular inflammation triggered by bacterial infection in mouse brain. LPS has been shown to induce tissue injury by damaging the nutritive microcirculation and by stimulating the release of cytokines [27]. HO-1 may protect against LPS-mediated injury by attenuating the expression of inflammatory mediators and improving microvascular perfusion. In the present study, we found that pre-induction of HO-1 attenuate LPS-induced expression of COX-2 protein and therefore decreased PGE_2 _accumulation. The role of HO-1 over-expression in COX-2 induction as well as PGE_2 _synthesis induced by various stimuli remains to be investigated.

Bernardini et al. [16] have shown that levels of HO-1, Hsp70, and Egr-1 proteins are increased within 4-15 h after LPS (10 μg/ml) administration in porcine aortic endothelial cells (pAEC). LPS (10 μg/ml, 15 h) also increases apoptosis rates about 4-7 folds in pAEC. Because HO-1 plays a protecting role in stress conditions, it is reasonable that 10 μg/ml of LPS damaged the pAEC and also induced HO-1 protein expression to defend against the injury insults. In our study, there was no COX-2 protein expression induced by LPS at 25 μg/ml for 24 h. The required concentration of LPS to induce significant COX-2 protein expression in bEnd.3 cells was a concentration of 100 μg/ml for 24 h. At this concentration, neither was cell damage observed nor HO-1 expression increased in bEnd.3 cells. This discrepancy may be due to different experimental conditions or to cell specificity.

Carbon monoxide (CO) is one of the main metabolites of heme degradation by HO-1 [28,29]. Its anti-inflammatory, anti-apoptotic and cytoprotective properties are well documented in different experimental models [15]. There is growing evidence to demonstrate the role of CO as anti-inflammatory and cytoprotective functions of HO-1 in various cell types [30-32]. The HO/CO signaling pathway plays an important role in host defense mechanisms, including inhibition of TLR4 signaling stimulated by LPS [33-35]. In the present study, LPS-induced COX-2 expression and PGE_2 _production was inhibited by pretreatment with either CoPP or CO-RM2. This inhibitory effect was reversed by pretreatment with ZnPP, an HO-1 activity inhibitor, and hemoglobin, a chelator of CO, suggesting that the inhibitory effect of HO-1 on COX-2 expression was partially mediated through CO. Consistent with our report, CO has been shown to down-regulate LPS-induced COX-2 expression and PGE_2 _secretion by inhibiting CCAAT/enhancer-binding protein (C/EBP) in LPS-treated RAW 264.7 cells [36]. In addition to CO, biliverdin and bilirubin have been reported to protect cells from the insult of oxidative stress in HO-1 siRNA-transfected HT22 cells [37]. The roles of biliverdin and bilirubin on LPS-induced COX-2 expression needs further investigating.

The transcription factor NF-κB is a major mediator of LPS signaling [38]. LPS-activated NF-κB is an important transcription factor for expression of inflammatory proteins. NF-κB is a downstream component of tyrosine phosphorylation. LPS has been shown to activate NF-κB through TLR4 leading to the expression of COX-2 [22]. It has also been reported that the attenuation of NF-κB activation by CoPP displays anti-inflammatory effects of HO-1 in various cell types [26]. Interestingly, CO has been reported to modulate several transcription factors, including NF-κB [30,31]. In the blood/vascular system, pretreatment of human umbilical vein endothelial cells (HUVEC) with CO-RM2 attenuates the LPS-induced activation of NF-κB [32]. Recently, CO has also been shown to block LPS-induced initial inflammatory response through inhibition of NF-κB in human monocytes [35]. In the present study, we found that LPS-induced COX-2 expression and p65 activation was inhibited by pretreatment with CoPP and CO-RM2, suggesting that induction of HO-1/CO inhibits LPS-induced COX-2 by inhibition of NF-κB in murine celebral endothelium.

In addition to NF-κB inhibition by HO-1/CO, other transcription factors, such as CCAAT/enhancer-binding protein (C/EBP) expression are decreased by HO-1/CO in LPS-treated RAW 264.7 cells [36] and activating protein (AP)-1 is also suppressed by hemin, an HO-1 inducer, in soleus muscles of Sprague-Dawley rats [39]. It is interesting that early growth response-1 (Egr-1) protein, which is a key transcription factor in regulating the inducible expression of microsomal PGE synthase 1 (mPGES-1) and therefore promotes PGE_2 _release [40], is also significantly reduced by an HO-1 inducer, CoPP, in IL-1β-treated chondrocytes [41]. All of NF-κB, C/EBP, AP-1, and Egr-1 transcription factors are involved in PGE_2 _synthesis catalyzed by induction of either COX-2 or mPGES-1. The activation of these transcription factors is suppressed by induction of HO-1 which exerts anti-oxidative and anti-inflammatory effects in several cell types [42], including endothelial cells [43]. We therefore hypothesized that there is a common mechanism in attenuation of these inflammation-related transcription factors by induction of HO-1/CO in these cells.

TLRs function as primary sensors of pathogens, which activate signaling pathways leading to the expression of cytokines [44]. It has been demonstrated that the signaling pathways are initiated by LPS binding to the TLR4 in endothelial cells [23]. Inflammatory signaling initiates when LPS binds to LPS-binding protein, which presents LPS to CD14. Binding of LPS to CD14 activates TLR4. Activation of TLR4 triggers the recruitment of myeloid differentiation factor (MyD88) and initiates NF-κB signaling pathway, leading to expression of inflammatory target proteins. In the present study, pretreatment with CO-RM2 attenuated the association between TLR4 and MyD88 (Figure 5D). CO-RM2 inhibited LPS-induced activation of NF-κB, which may be due to attenuation of TLR4/MyD88 signaling, consistent with results indicating that CO suppresses TLR4/MyD88 signaling in murine macrophages [45].

COX-2 has been suggested to contribute to LPS-induced cellular damage [3]. It has been reported that pretreatment with a selective COX-2 inhibitor protects neuronal cultures from LPS-induced cytotoxicity through attenuation of COX-2 expression and PGs synthesis [46]. Interestingly, COX-2-selective inhibitors have been reported to induce HO-1 expression in various cell types [47,48]. These reports suggest that the protective effects of COX-2 inhibitors may be due to HO-1 induction [49]. Furthermore, it has been also reported that the COX-2 metabolite, PGE_2_, induces HO-1 through PKA and PI3K signaling pathways via EP2 receptors in C6 cells [50]. Induction of HO-1 may be a defensive response initiated by PGE_2 _and serve a critical role for a cytoprotective mechanism during oxidative stress. There seems to be an interesting interactive relationship between COX-2 and HO-1, that is, over-expression of COX-2 associated with PGE_2 _release induces HO-1 expression which in turn inhibits COX-2 expression and therefore diminishes PGE_2 _production. The relationship between HO-1 and COX-2 is an interesting issue and needs detailed investigation.

**Figure 6:**
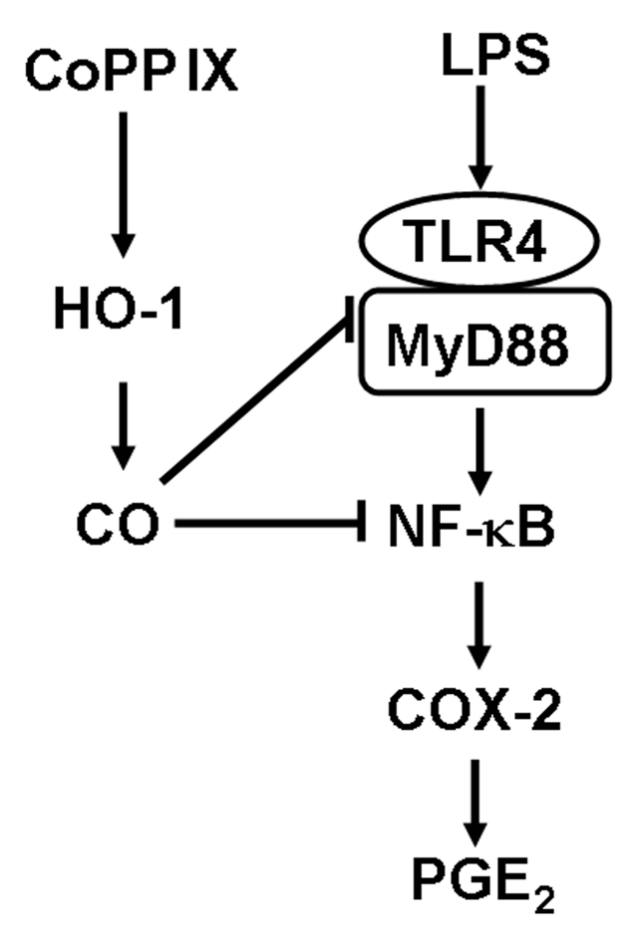
**Down-regulation of LPS-induced COX-2 expression and PGE_2 _production by HO-1/CO in bEnd.3 cells**. LPS-induced COX-2 expression and PGE_2 _production were mediated by a TLR4/MyD88/NF-κB pathway. Induction of HO-1 by pretreatment with CoPP or infection with Adv-HO-1 attenuated LPS-induced COX-2 expression and PGE_2 _production in bEnd.3 cells. A HO-1 by-product, CO, at least in part, attenuated LPS-induced COX-2 expression through interruption of TLR4/MyD88/NF-κB pathway in bEnd.3 cells.

As depicted in Figure 6, this study demonstrated that up-regulation of HO-1 inhibits LPS-mediated COX-2 expression and PGE_2 _synthesis in murine brain endothelial cells (bEnd.3). Treatment of bEnd.3 cells with LPS induces COX-2 protein and PGE2 via Toll-like receptor 4-mediated activation of NF-κB. However, pharmacologic induction or gene transfer of HO-1 blocks LPS-mediated COX-2 expression and activity. This inhibitory effect of HO-1 on COX-2 expression was mediated through retardation of NF-κB and could be reversed by an HO inhibitor, ZnPP, or a CO-scavenger, hemoglobin. Moreover, the exogenous administration of the CO releasing molecule, CO-RM2, mimics the effect of HO-1.

## Conclusions

Our results show strong evidence that overexpression of HO-1/CO exerts an inhibitory effect on LPS-induced COX-2/PGE_2 _synthesis in cerebrovascular endothelial cells. Our results reinforce the emerging role of cerebrovascular endothelium-derived HO-1 as a protector for preventing cerebral vascular inflammation triggered by bacterial infection.

## Competing interests

The authors declare that they have no competing interests.

## Authors' contributions

RHS designed and performed experiments, acquisition and analysis of data, and drafted the manuscript. CMY has conceived of the study, participated in its design and coordination, has been involved in drafting the manuscript and revising it critically for important intellectual content and have given final approval of the version to be published. The authors have read and approved the final version of this manuscript.

## Author Details

Department of Physiology and Pharmacology, Chang Gung University, Tao-Yuan, Taiwan
